# Genipin Inhibits Mitochondrial Uncoupling Protein 2 Expression and Ameliorates Podocyte Injury in Diabetic Mice

**DOI:** 10.1371/journal.pone.0041391

**Published:** 2012-07-19

**Authors:** Wenjing Qiu, Yang Zhou, Lei Jiang, Li Fang, Lu Chen, Weifang Su, Ruoyun Tan, Chen-yu Zhang, Xiao Han, Junwei Yang

**Affiliations:** 1 Center for Kidney Disease, 2nd Affiliated Hospital, Nanjing Medical University, Nanjing, Jiangsu, China; 2 Jiangsu Diabetes Center, State Key Laboratory of Pharmaceutical Biotechnology, School of Life Science, Nanjing University, Nanjing, Jiangsu, China; 3 Key Laboratory of Human Functional Genomics of Jiangsu Province, Nanjing Medical University, Nanjing, Jiangsu, China; The University of Manchester, United Kingdom

## Abstract

Diabetic nephropathy (DN) is one of the most common causes of end stage renal disease (ESRD) in China, which requires renal replacement therapy. Recent investigations have suggested an essential role of podocyte injury in the initial stage of DN. This study investigated the potential therapeutic role of genipin, an active extract from a traditional Chinese medicine, on progression of DN in diabetic mice induced by intraperitoneally injection of streptozocin (STZ). In diabetic mice, orally administration of genipin postponed the progression of DN, as demonstrated by ameliorating body weight loss and urine albumin leakage, attenuating glomerular basement membrane thickness, restoring the podocyte expression of podocin and WT1 in diabetic mice. The protective role of genipin on DN is probably through suppressing the up-regulation of mitochondrial uncoupling protein 2 (UCP2) in diabetic kidneys. Meanwhile, through inhibiting the up-regulation of UCP2, genipin restores podocin and WT1 expression in cultured podocytes and attenuates glucose-induced albumin leakage through podocytes monolayer. Therefore, these results revealed that genipin inhibited UCP2 expression and ameliorated podocyte injury in DN mice.

## Introduction

One hundred million or 9.7 percent of Chinese adult residents had diabetes in 2010. Between 9% and 36% of patients with diabetes ultimately develop diabetic nephropathy (DN), which in China is the third most common cause of end stage renal disease (ESRD) requiring renal replacement therapy [1], [2], [3], [4], [5]. The clinical manifestation of DN is characterized by persistent albuminuria and proteinuria, thickness of glomerular basement membrane (GBM), accumulation of extra-cellular matrix (ECM), which results in glomerulosclerosis and tubulointerstitial fibrosis and progressive renal dysfunction [6], [7], [8], [9], [10]. Traditionally, alterations of mesangial cells were the focus of investigations in deciphering mechanisms of DN. However, recent renal biopsy studies in human and mice have provided evidence suggesting that podocyte injury were evident early in the progression of DN [9], [11], [12].

Podocytes, a kind of highly and terminal differentiated cells, extend foot processes toward GBM, which form the slit diaphragm among adjacent podocytes. The integrity of slit diaphragm determinates the permeability of glomerular filtration barrier. It has been reported that foot process widening, as well as podocyte number and density reduction were commonly evident in both type 1 and type 2 diabetes [8], [9], [13], [14], [15], [16]. In this study, ahead of renal dysfunction, podocyte injuries demonstrated by persistent albuminuria, foot process effacement and podocyte markers loss were evident in kidneys of diabetic mice, suggesting an essential role of podocyte injury in the pathogenesis of DN. Current therapies towards DN in clinical settings are largely symptomatic treatment including lower blood glucose level, control cholesterol and blood pressure and prevent complications [17], [18], [19], [20], [21], [22], [23]. New therapeutic approaches remain urgent to halt the progression of DN in patients.

Genipin is a glycone derived from an iridoid glycoside called geniposide present in fruit of gardenia jasminoides, which has been used over hundreds of years in traditional Chinese medicine to alleviate symptoms of diabetes. Zhang et al. found that genipin could stimulate insulin secretion by pancreatic β-cells, however, this effect was dependent on the presence of a mitochondrial membrane protein, uncoupling protein 2 (UCP2) [24]. UCP2 is a mitochondrial inner membrane carrier protein expressed in a number of tissues, including pancreatic islets and kidney. UCP2 mediated proton leakage decreases the yield of ATP from glucose and consequently inhibits glucose-stimulated insulin secretion [25]. As an inhibitor of UCP2 function, genipin helps lowering blood glucose by stimulating insulin secretion [24]. However, as an alternative medicine already applied in clinical settings, whether genipin protect podocyte injury induced by glucose and its underlying mechanism remains unknown.

In this study, we investigated the potential therapeutic role of genipin on progression of DN in diabetic mice induced by intraperitoneally injection of streptozocin (STZ). Our results suggested that orally administration of genipin significantly ameliorates urinary albumin excretion, glomerular basement membrane (GBM) thickness and podocyte injury in diabetic mice. Of note, inhibition of the UCP2 expression by genipin plays an essential role in halting the progression of DN.

## Results

### Genipin ameliorates body weight loss and urine albumin leakage in diabetic mice

**Figure 1:**
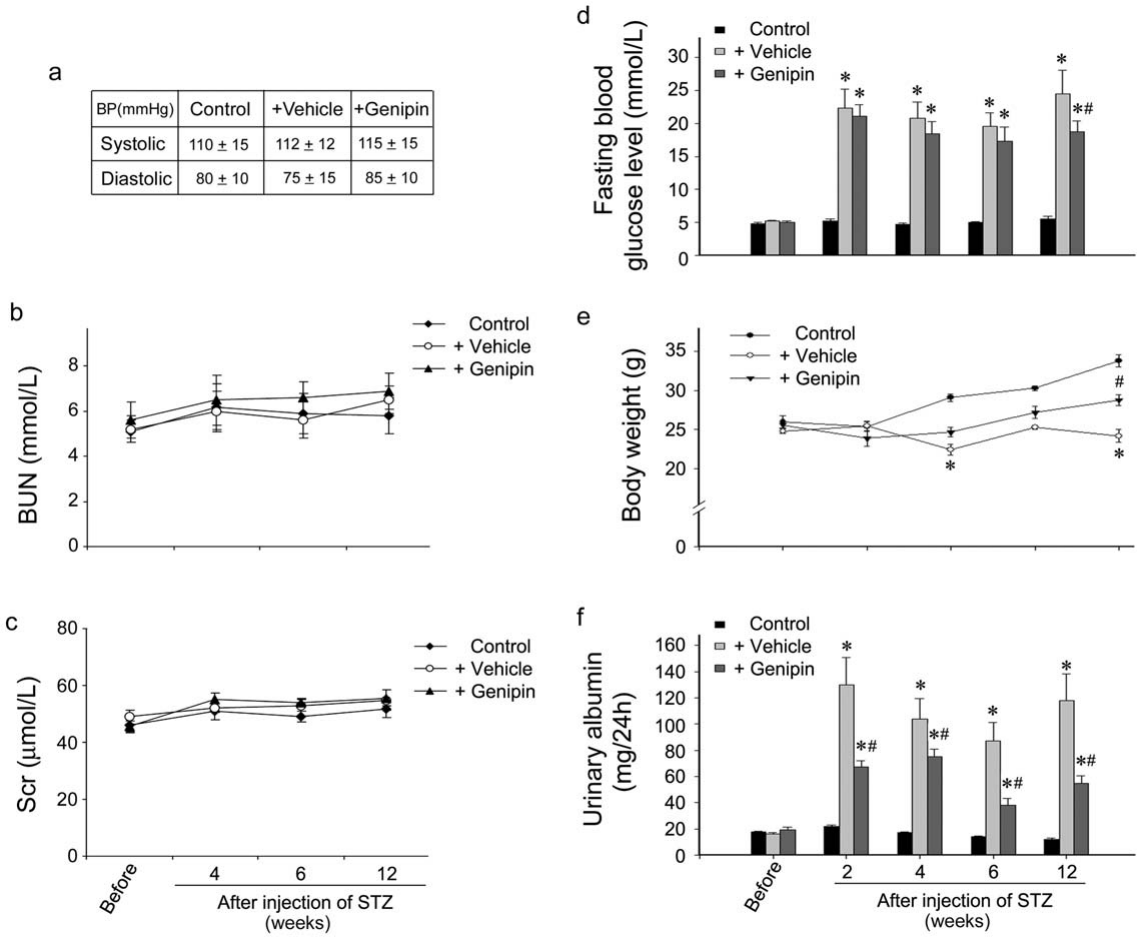
Genipin ameliorates body weight loss and urine albumin leakage in diabetic mice. (a) Blood pressure (BP) of control group, diabetes group treated with vehicle and diabetes group treated with genipin for 12 weeks were measured by a non-invasive blood pressure analysis system (Softron, BP-98A). Values (mmHg) are means ± SE from 6 animals/group. BUN (b) and Scr (c) of control group, diabetes group treated with vehicle and diabetes group treated with genipin were examined in experimental period. Values are means ± SE from 6 animals/group. (d) Fast blood glucose levels of control group, diabetes group treated with vehicle and diabetes group treated with genipin were examined in experimental period. Values (mmol/L) are means ± SE from 6 animals/group at each time point. *P<0.05 vs. sham-control, #P<0.05 genipin vs. vehicle. (e) Body weight of control group, diabetes group treated with vehicle and diabetes group treated with genipin were examined in experimental period. Values (g) are means ± SE from 6 animals/group at each time point. *P<0.05 vs. sham-control, #P<0.05 genipin vs. vehicle. (f) Urine albumin of control group, diabetes group treated with vehicle and diabetes group treated with genipin were examined in experimental period. Values (mg/24h) are means ± SE from 6 animals/group at each time point. *P<0.05 vs. sham-control, #P<0.05 genipin vs. vehicle.

The blood pressure (BP), blood urine nitrogen (BUN) and serum creatinine (Scr) did not show markedly changes after 12 weeks in both DN groups (Fig. 1a through 1c). Hyperglycemia and body weight loss are two major clinical manifestations of type 1 diabetes. Therefore, we examined blood glucose level and body weight in diabetic mice induced by STZ (Fig. 1d and 1e). As compared with control group, the blood glucose level of vehicle treated group markedly increased as early as two weeks after STZ injection and persistent until the end of the experimental period (12 weeks). Although the blood glucose level of genipin treated group appeared to be lower than vehicle treated group, they were all higher than the diagnostic criteria of diabetes (fasting blood glucose level >11.1mmol/L or random blood glucose level >20.0 mmol/L) and there were no statistically significant difference between both diabetic groups (Fig. 1d). As to the body weight, the vehicle treated diabetic mice showed a sustained reduction of body weight over 12 weeks when compared with the normal controls. However, genipin treatment greatly ameliorated the body weight loss (Fig. 1e). We next investigated the urine albumin leakage, early markers of diabetic nephropathy. Administration of genipin resulted in a substantial alleviation of albuminuria in diabetic mice. As shown in Fig. 1f, urine albumin levels markedly increased since 2 weeks after STZ. Administration of genipin reduced albumin excretion by approximately 50%.

### Genipin attenuates glomerular basement membrane thickening and podocyte foot process effacement

**Figure 2:**
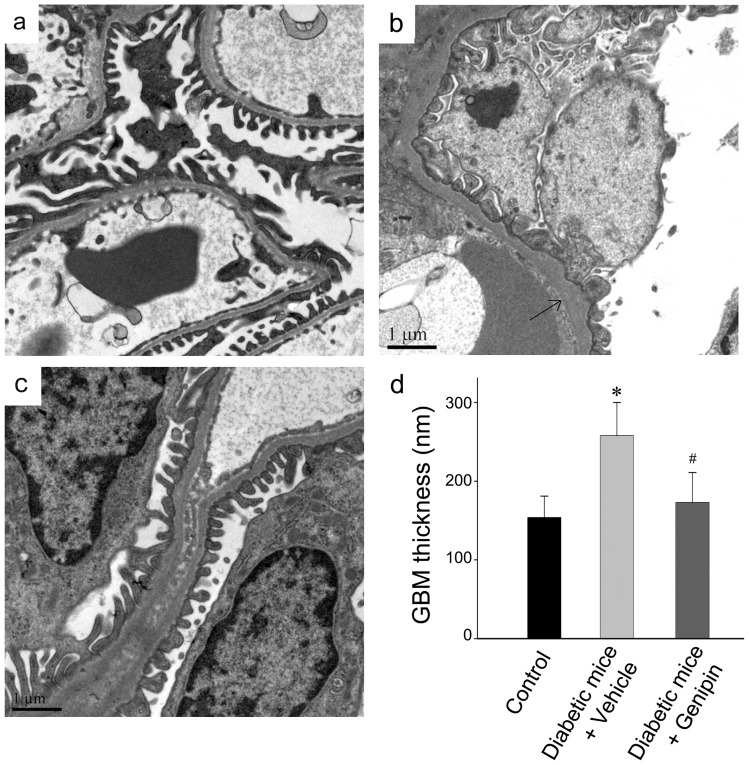
Genipin attenuates glomerular basement membrane thickening and podocyte injury. (a–c) Representative electron micrographs show GBM thickening and foot process effacement in the vehicle treated diabetic kidney. These morphologic injuries were attenuated in diabetic mice that received genipin. Scale bar, 1μm. (a) sham-control. (b) Diabetic mice with vehicle. (c) Diabetic mice with genipin. (d) Graphic presentation of the GBM thickness in each group. The data were calculated based on individual values determined on ten fields per mouse, six mice per group (n = 6). Values (nm) are means ± SE. *P<0.05 vs. sham-control, #*P*<0.05 genipin vs. vehicle.

Fig. 2 demonstrates that genipin attenuates glomerular basement membrane (GBM) thickening and podocyte injury, both of which are initial pathological changes that result in albuminuria. Compared with normal control, electron microscopy exhibited thickening of GBM and foot process effacement in the vehicle treated diabetic kidney. These morphologic injuries were attenuated in diabetic mice that received genipin (Fig. 2c and 2d). In addition, electron microscopy showed denuded GBM in several diabetic kidneys (data not shown), which probably suggested a loss of podocyte.

### Genipin restores the expression and distribution of podocin and WT1 in diabetic mice

**Figure 3:**
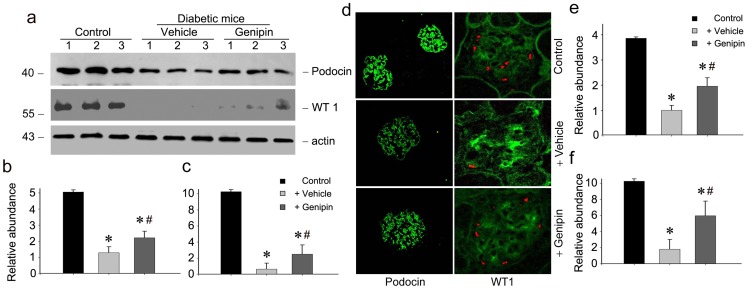
Genipin restores the expression of podocin and WT1 in diabetic mice. (a) Western blot analysis shows the results of podocin and WT1 in the kidneys of each group. The samples were reprobed with actin to confirm equal loading of each lane. Representative pictures show the results of three animals per group. (b) Graphic presentation of relative podocin abundance normalized to actin. *P<0.05 vs. control, #*P*<0.05, genipin vs. vehicle. (c) Graphic presentation of relative WT1 abundance normalized to actin. *P<0.05 vs. control, #*P*<0.05, genipin vs. vehicle. (d) Immunofluorescent staining of podocin (green) and WT1 (red) protein in each group, respectively. Collagen IV was stained green to localize WT1 (red). The representative figures after three repetitions were shown. (e) Graphic presentation of relative fluorescent intensity of podocin. *P<0.05 vs. control, #*P*<0.05, genipin vs. vehicle. (f) Graphic presentation of relative fluorescent intensity of WT1. *P<0.05 vs. control, #*P*<0.05, genipin vs. vehicle.

Podocin is a protein which lines the podocytes and assists in maintaining the construction of filtration barrier. Wilms' tumor 1 gene (WT1) is expressed in the podocytes as a cell specific marker. As a kind of terminal differentiated cells, injury of podocytes is characterized by the loss of podocin and WT1 expression, which ultimately results in cell detachment and loss. Therefore, the expression and deposition of podocin and WT1 were examined. Fig. 3 shows the expression of podocin in kidneys of diabetic mice determined by western blot (Fig. 3a) and immunofluorescent staining (Fig. 3d). The expression of podocin protein was markedly decreased in vehicle treated diabetic kidneys. However, restoration of podocin expression was evident in genipin treated diabetic kidneys. Similar results were observed for renal WT1 expression. Both Western blot analyses of whole kidney lysates and immunofluorescent staining exhibited a restoration of WT1 expression in genipin treated group, which is consistent with the notion that genipin restored the podocyte injury in DN mice.

### Genipin suppresses the up-regulation of UCP2 protein in diabetic kidneys

**Figure 4:**
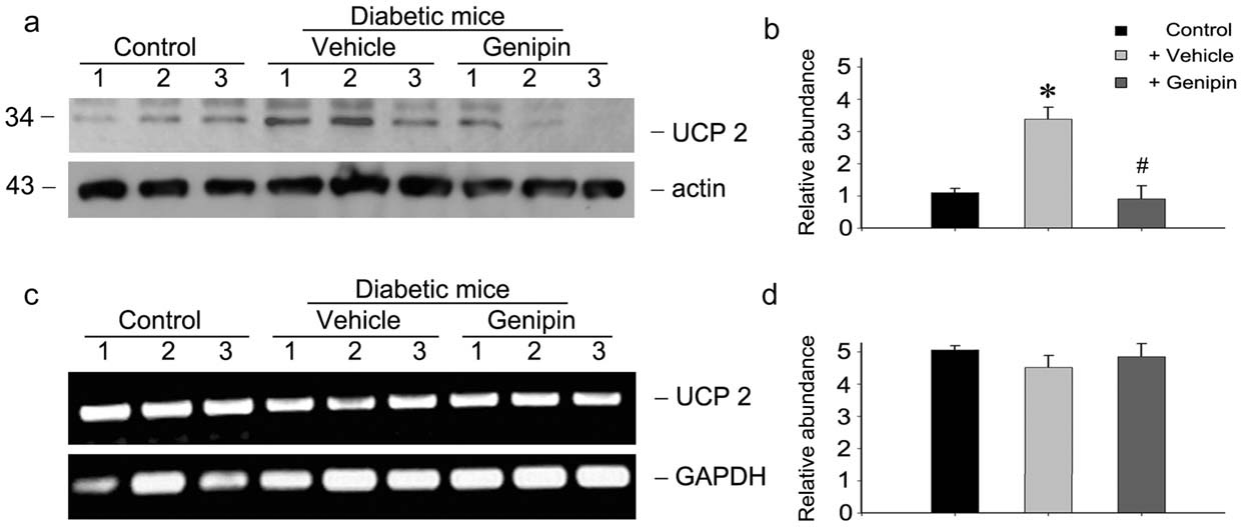
Genipin suppresses the upregulation of UCP2 protein expression in diabetic kidneys. (a) Western blot analysis shows the results of UCP2 in the kidneys of each group. The membrane was reprobed with actin to confirm equal loading of each lane. Representative pictures show the results of three animals per group. (b) Graphic presentation of relative UCP2 abundance normalized to actin. *P<0.05 vs. control, #*P*<0.05, genipin vs. vehicle. (c) RT-PCR analysis shows the results of UCP2 mRNA in the kidneys of each group. Representative pictures show the results of three animals per group. (d) Graphic presentation of relative UCP2 mRNA abundance normalized to actin.

The therapeutic effect of genipin on podocyte injury is discernible. However, the underlying mechanism remains unsolved. As the target of genipin, mitochondrial uncoupling protein 2 (UCP2) expression was examined. As shown in Fig. 4a, western blot analysis demonstrates that UCP2 protein was up-regulated in vehicle treated diabetic mice. Administration of genipin markedly inhibited the upregulation of UCP2 protein in diabetic kidneys. However, it seems that UCP2 mRNA expression in the kidney was unaffected after diabetes in the presence or absence of genipin (Fig. 4c). These results suggested that genipin inhibited UCP2 protein expression in diabetic kidneys.

### Genipin restores podocin and WT1 expression in cultured podocytes

**Figure 5:**
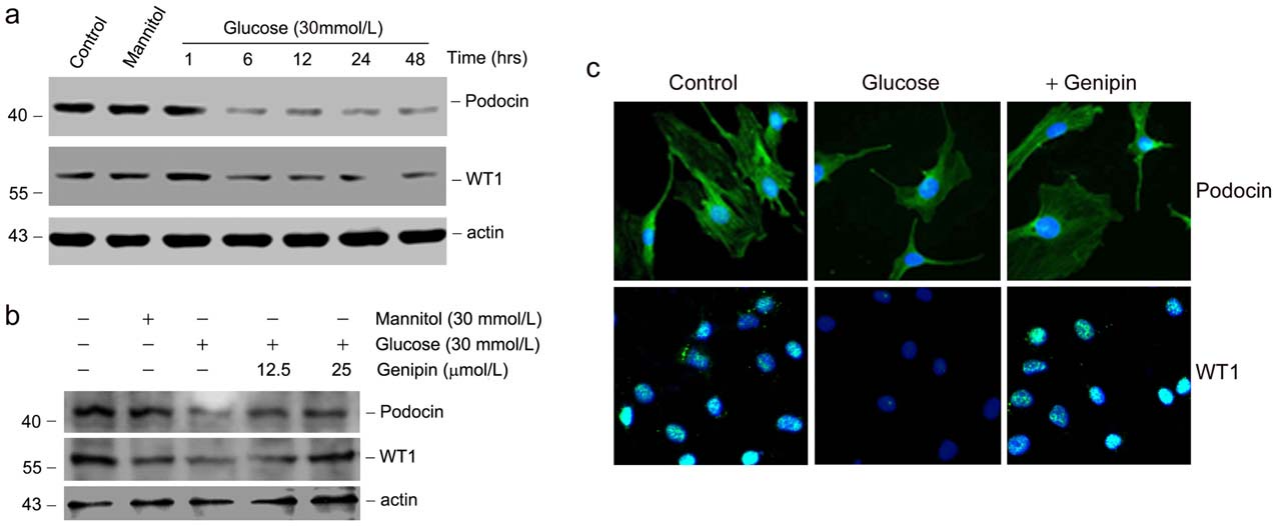
Genipin restores podocin and WT1 expression in cultured podocytes. (a) Western blot analysis shows the results of podocin and WT1 in podocytes incubated with D-Glucose for different time periods as indicated. The membrane was reprobed with actin to confirm equal loading of each lane. (b) Western blot analysis shows the results of podocin and WT1 in D-Glucose (24 h) incubated podocytes pre-treated (0.5 h) with or without genipin. The membrane was reprobed with actin to confirm equal loading of each lane. (c) Immunofluorescence staining of podocin and WT1 (green) protein, respectively. Cell nuclei were stained with DAPI (blue).

Given that genipin protects podocyte injury in DN, we next examined the effects of genipin on glucose-induced podocyte injury using an *in vitro* podocyte culture system. After incubation with 30mmol/L of D-glucose, the expression of podocin and WT1 were both markedly decreased. Time-dependent study revealed that podocin and WT1 expression was down-regulated as early as 6 hours' incubation, consistent with the clinical manifestation that podocyte injury is one of the initial events during diabetic nephropathy (Fig. 5a). As to investigate the protective effects of genipin, mouse podocytes were incubated with D-glucose in the presence or absence of genipin, and podocin as well as WT1 expression were examined. As shown in Fig. 5b, western blot analysis showed that podocin and WT1 expression were markedly decreased after glucose treatment. However, pre-treatment with genipin restored podocin and WT1 expression. Of note, the restoration of podocin and WT1 expression was genipin dose-dependent. Similar results were also obtained in immunofluorescent staining assay (Fig. 5c).

### Genipin reduces UCP2 expression induced by high glucose in podocytes

**Figure 6:**
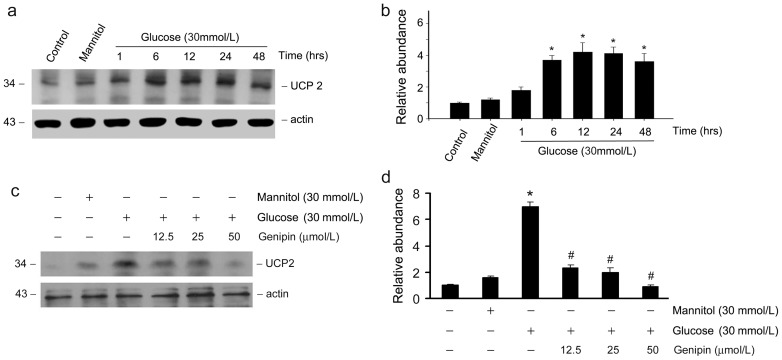
Genipin reduces UCP2 expression induced by high glucose in podocytes. (a) Western blot analysis shows the results of UCP2 in podocytes incubated with D-Glucose for different time periods as indicated. The membrane was reprobed with actin to confirm equal loading of each lane. (b) Graphic presentation of relative UCP2 abundance normalized to actin. *P<0.05 vs. control. *n* = 3. (c) Western blot analysis shows that pre-incubation with genipin inhibited glucose-induced UCP2 expression. The membrane was reprobed with actin to confirm equal loading of each lane. (d) Graphic presentation of relative UCP2 abundance normalized to actin. *P<0.05 vs. control. #*P*<0.05 vs. glucose incubated cells without genipin pre-treatment. *n* = 3.

**Figure 7:**
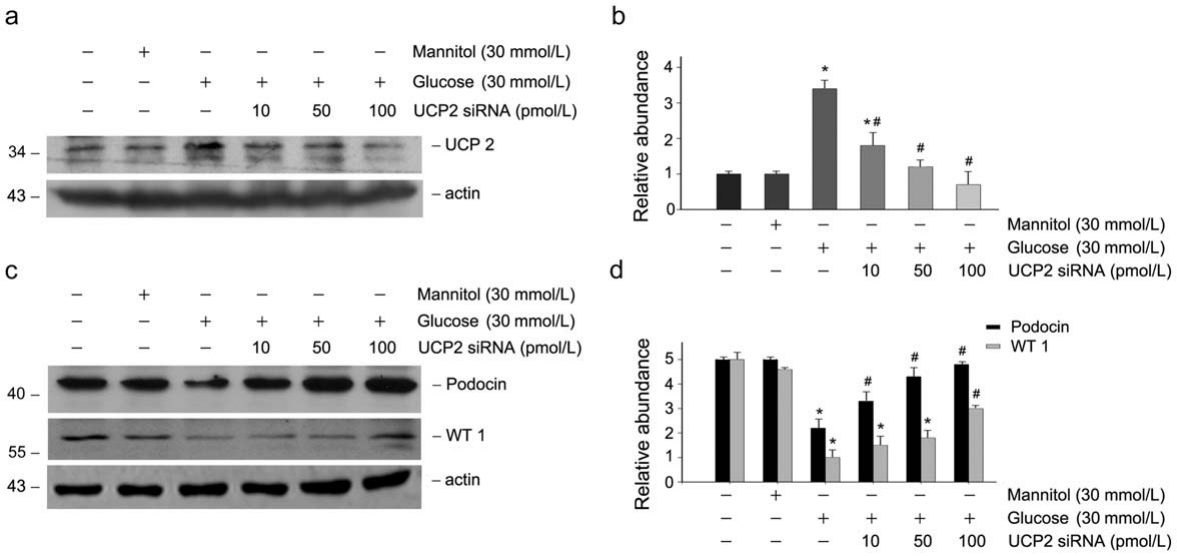
Downregulation of UCP2 by RNA interference restores podocin and WT1 expression depressed by high glucose in podocytes. (a) Western blot analysis shows the results of UCP2 in podocytes transfected with different concentrations of UCP2 siRNA as indicated. The membrane was reprobed with actin to confirm equal loading of each lane. (b) Graphic presentation of relative UCP2 abundance normalized to actin. *P<0.05 vs. control. #*P*<0.05 vs. glucose incubated cells transfected with control siRNA. *n* = 3. (c) Western blot analysis shows the results of podocin and WT1 in podocytes transfected with different concentrations of UCP2 siRNA as indicated. The membrane was reprobed with actin to confirm equal loading of each lane. (d) Graphic presentation of relative podocin and WT1 abundance normalized to actin. *P<0.05 vs. control. #*P*<0.05 vs. glucose incubated cells transfected with control siRNA. *n* = 3.

We further investigated the regulation of UCP2 expression by genipin in cultured podocytes. As shown in Fig. 6a, western blot analysis showed a markedly increased expression of UCP2 protein after glucose incubation. Time-dependent study revealed that UCP2 protein was significantly increased as early as 6 hours of glucose incubation. Pre-incubation with genipin, however, inhibited glucose-induced UCP2 expression (Fig. 6c). Likewise, genipin reduced UCP2 expression induced by glucose in a genipin dose-dependent manner (Fig. 6d). To verify the protective role of down-regulation of UCP2 on glucose-induced podocyte injury, RNA interference approach was applied to inhibit UCP2 expression induced by glucose treatment. Fig. 7a shows that more than 75% repression of UCP2 protein was detected in podocytes transfected with 100pmol of UCP2 siRNA. To investigate the protective effects after downregulation of UCP2 expression, podocytes were transfected with negative control or UCP2 siRNA before incubation with glucose, podocin and WT1 expression were thereafter examined. Western blot analysis shows that glucose markedly decreased podocin and WT1 expression in podocytes. However, pre-transfected with UCP2 siRNA largely restored podocin and WT1 expression (Fig. 7c). At a dose of 100pmol, UCP2 siRNA could almost totally abolish glucose-induced depression of podocin and WT1 in podocytes (Fig. 7d).

### Genipin attenuates high glucose induced filtration barrier dysfunction of podocytes

**Figure 8:**
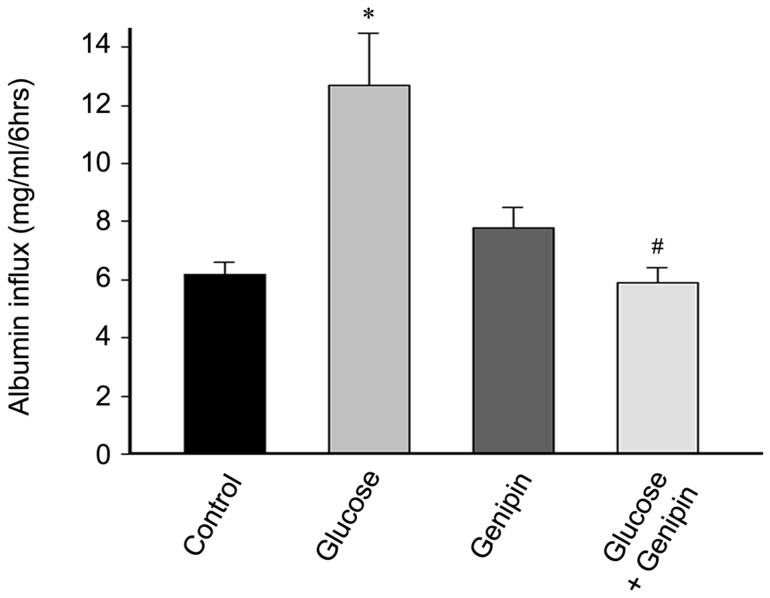
Genipin attenuates high glucose induced filtration barrier dysfunction of podocytes. Graphic presentation shows the albumin flux rate across the differentiated podocyte monolayer in each group after 6 hours' treatment. Values (mg/ml) are means ± SE. *P<0.05 vs. control, #*P*<0.05 genipin vs. glucose.

Podocyte is one of the components of renal filtration barrier. Podocyte injury is one of the causes of albuminuria, the initial clinical manifestation of diabetic nephropathy. To examine the functional consequence of glucose-induced podocyte injury *in vitro*, we examined the filtration barrier function of podocyte using a cellular permeability influx assay system. The albumin flux rate across the differentiated podocyte monolayer was measured. Differentiated podocytes in the presence or absence of genipin were incubated with or without glucose (30mmol/L) for 6 hours to induce podocyte injury; the media were collected and subjected to albumin assay. As shown in Fig. 8, compared with the controls, high glucose incubation resulted in a marked albumin influx across the podocyte monolayer. However, genipin significantly attenuated high glucose induced albumin influx. This result suggests that genipin attenuated glucose induced filtration barrier dysfunction of podocyte monolayer.

## Discussion

Diabetic nephropathy (DN) is a major complication of diabetes mellitus, which becomes one of the leading causes of end-stage renal disease (ESRD) [26], [27], [28], [29]. Despite of the traditional focus of mesangial cells in deciphering molecular mechanisms of DN [27], [30], [31], accumulated evidence indicates that injury of terminally differentiated podocytes in the initial stage of DN will result in massive proteinuria, renal insufficiency and irreversible renal failure [8], [9], [13], [15], [32], [33], [34], [35], [36]. The present study showed that oral administration of genipin partially prevented the progression of DN induced by STZ injection. It reduced the production of albuminuria and mitigated podocytes lesions. In addition, our results suggest that genipin might protect hyperglycemia-induced podocytes injury by inhibition of mitochondrial uncoupling protein 2 (UCP2).

Over the past decade, renal mesangial and GBM have attracted most attentions in DN-related investigations. Although the classical initial hallmarks of DN are glomerular hypertrophy, mesangial matrix expansion and GBM thickness, investigations in diabetic patients and animals revealed that podocyte injuries attribute to the onset of albuminuria [9]. The podocyte lesions, including foot process effacement, hypertrophy, detachment, apoptosis and epithelial-to-mesenchymal transition (EMT), are all well-documented characteristics of DN [10], [13], [32]. In this study, hyperglycemia-induced podocyte injury is demonstrated as urine albumin leakage, foot process effacement and loss of podocyte markers, podocin and WT1. In the ex vivo cell culture system, the podocyte monolayer might not as confluent as in vivo which could completely block the filtration of albumin, however, compared with controls, the markedly increased albumin influx after glucose treatment suggested impaired podocyte barrier. Administration of genipin, a traditional Chinese herb extract, has been practiced in clinical settings for many years. However, its pharmacologic mechanism remains largely unknown. This investigation suggested that in addition to stimulation of insulin, genipin might protect the integrity of podocyte and delay the onset of DN through inhibition of UCP2 protein expression in diabetes.

Genipin, a Chinese herb extract from *Gardenia jasminoides Ellis* fruits, has been used for centuries in traditional Chinese medicine to relieve symptoms of type 2 diabetes. Zhang et al demonstrated that genipin could reverse glucose- and obesity-induced β cell dysfunction and stimulate pancreatic islets to secret insulin [24]. However, its protective effort on renal injury remains largely unknown. In this study, we found that DN was characterized by albuminuria, thickness of GBM, as well as glomerular hypertrophy and interstitial fibrosis (data not shown) in wild-type mice. Not as sensitive as Balb/c or CD-1 mice (∼300mg/d), the urinary albumin levels of wild-type mice after DN were ∼100mg/d, so was GBM thickness; however, both urinary albumin extraction level and GBM thickness were significantly increased as compared to sham-control mice. Administration of genipin significantly reduced urine albumin excretion, podocytes loss and glomerular basement membrane (GBM) thickness, which are early symptoms and pathologic changes of DN. However, the renal protective role of genipin against DN seems to be independent of the blood glucose levels. This might be attributed to the STZ-induced hyperglycemia animal models we applied. STZ injection directly and irreversibly destroys pancreatic islets β cell, which made the secretion stimulation effect of genipin unavailable. Genipin has already been administered as one of the therapeutic strategies for type 2 diabetes in clinical settings of traditional Chinese medicine. The therapeutic effects of genipin on type 2 diabetes were presumed to be attributed to its role on stimulating insulin secretion and thereafter modulating the blood glucose level. No evidence to date suggested that genipin could increase insulin sensitivity. However, it has been identified that hyperinsulinemia and insulin resistance were the major pathophysiologic conditions of type 2 diabetes but not lack of insulin as previously considered. We hypothesis that the effectiveness of genipin on ameliorating the symptoms of both type 1 and type 2 diabetes might probably independent of insulin and blood glucose level, whose underlying mechanism needs further investigation.

UCP2 is a mitochondrial carrier protein, which is expressed in various tissues including kidney, heart, pancreatic islets, et al. It was reported that through mediating proton leakage, UCP2 reduces the yield of ATP from glucose and inhibits glucose-stimulated insulin secretion [25]. Zhang et al. suggested that oxidative stress-activated, UCP2-mediated mitochondrial proton leak can be inhibited by genipin treatment, consequently increase insulin secretion and reverse β cell dysfunction [24]. However, the unknown from these studies is how genipin inhibits UCP2. In this study, it is demonstrated that the amount of UCP2 protein expression was up-regulated in the kidney from DN mice and genipin treatment inhibited this up-regulation both *in vivo* and *in vitro*. On the contrary, the expression of UCP2 mRNA remained unchanged, which suggested that genipin negatively regulated UCP2 by inhibiting its protein expression. As a mitochondrial protein, UCP2 was reported to regulate cell energy metabolism, oxidative stress, iron transportation, et al. Whether genipin affect these functions of UCP2 in diabetes needs further investigations. In addition, whether genipin inhibits UCP2 translation or increases UCP2 degradation needs further studies. Meanwhile, as the multiple functions of mitochondrial UCP2, how up-regulated UCP2 affects the podocyte pathophysiology, such as cell skeleton, cell cycles, which are probably the early events during DN progression remains unknown.

In summary, we have demonstrated herein that administration of genipin attenuates hyperglycemia-induced podocyte lesion and therefore delays the progression of diabetic nephropathy through negatively regulation of UCP2 protein expression. Although genipin has already been adopted in clinical settings as a part of alternative medicine for diabetic patients, more studies are needed to understand the mechanism for combating diabetic renal insufficiency.

## Methods

### Ethics statement

All of the following details of the study were approval by institutional review board of Nanjing Medical University.

### Animal model

Male C57BL/6J mice that weighted 18 to 22 g were obtained from Model Animal Research Center of Nanjing University. They were housed in the animal facilities of Center for Kidney Disease of Nanjing Medical University with free access to food and water and treated in compliance with the regulations and protocols of institutional review board of Nanjing Medical University. Mice were randomly divided into groups treated with streptozocin (STZ) (Sigma-Aldrich) or left untreated. STZ was dissolved in sterile citrate buffer (pH 4.5) and injected intraperitoneally (50mg/kg body weight) within 10 minutes of the preparation for 5 consecutive days. Diabetes mellitus was confirmed by measuring glucose levels tail venous blood using a reflectance meter (Omron). Mice with fasting blood glucose level >11.1mmol/L and random blood glucose level >20.0 mmol/L were included in experiments. Diabetic mice were randomly assigned into two groups (with 6 mice per group): vehicle treated and genipin treated. The genipin used in this study was obtained from Wako chemicals USA, Inc., whose purity is 98%+ (HPLC). Genipin was dissolved in polyethylene glycol (PEG, Sigma-Aldrich) and administered orally (50mg/kg body weight) daily. For the vehicle treated group, PEG was given. Periodically, fasting blood glucose, body weight were measured and urine samples for quantitative measurement of albuminuria were collected in metabolic cages. STZ treated mice were sacrificed at 12 weeks and the kidneys were removed. One part of the kidney was fixed in 2% glutaraldehyde, followed by epoxy resin embedding for electron microscopic studies. And the rest was snap-frozen in liquid nitrogen, and stored at −80°C for immunofluorescent staining and protein extraction.

Mice blood pressure (BP) was measures by a non-invasive blood pressure analysis system (Softron, BP-98A). Blood urea nitrogen (BUN) and serum creatinine (Scr) levels were evaluated using commercial kits (Bioassay Systems, DIUR-500, DICT-500). Diabetic nephropathy was characterized by albuminuria, thickness of GBM, as well as glomerular hypertrophy and interstitial fibrosis. Although not as sensitive as mice with other genetic background (Balb/c and CD-1), both urinary albumin extraction level and GBM thickness were significantly increased as compared to sham-control mice.

### Cell Culture and Treatment

Mouse conditionally immortalized podocyte cell line was cultured as described previously [15]. Briefly, podocytes were grown on collagen type I (Sigma) -coated culture dishes in RPMI 1640 medium supplemented with 10% fetal bovine serum (FBS), (Invitrogen), 100U/ml penicillin, 100U/ml streptomycin and 50U/ml mouse recombinant γ-interferon at 33°C. To induce differentiation, podocytes were cultured at 37°C in the absence of γ-interferon for 10–14 days before harvested for experiment. Podocytes grown under differentiating condition were seeded at 80% confluence in complete medium containing 10% FBS. After 24 hours, the cells were changed to serum-free medium and incubated for additional 16 hours. For high glucose treatment, cells were incubated with 30mmol/L of D-glucose (Sigma-Aldrich) pretreated (30 mins) with or without genipin for various periods of time as indicated, while control cells were treated with 30mmol/L of D-mannitol (Sigma-Aldrich) and the glucose concentration of control cell was 5mmol/L. UCP2 siRNA or negative control siRNA (Invitrogen) was transient transfected using Lipofectamine 2000 according to the instructions by the manufacturer (Invitrogen).

### Urine Albumin Assay

Urine albumin was measured using mouse Albumin ELISA quantitation kit, according to the protocols by the manufacturer (Bethyl Laboratories).

### Electron Microscopy

Electron microscopy of kidney tissue was carried out by conventional protocol described elsewhere [37]. Briefly, mouse kidney samples were fixed with 2.5% gluteraldehyde, 0.1M sodium cacodylate and 5mM calcium chloride (pH 7.2) at room temperature. Postfixation was completed with 1% OsO4 for 1 hour. Specimens were dehydrated in graded ethanols and infiltrated with 1:1 mixture of propylene oxide-Polybed 812 epoxy resin for 3 hour at room temperature, followed by a re-infiltration with pure epoxy resin overnight at 4°C. Samples were embedded and polymerized at 60°C for 48 to 72 hours. Ultrathin sections (60nm) were stained with 2% uranylacetate, followed by 1% lead citrate. Electron micrgraphs were taken with a Zeiss 910 Advanced Transmission Electron Microscope (Zeiss).

### Immunofluorescent staining

Indirect immunofluorescent staining was performed using an established protocol [38]. Briefly, embedded with OCT (Sakura), kidney cryosections were prepared at 5μm thickness and fixed for 5 minutes in PBS containing 3% paraformaldehyde. The sections were blocked with 2% bovine serum albumin in PBS for 30 minutes, and then incubated with primary antibodies against podocin (Sigma), WT1 (Santa Cruz, California, USA) and Collagen IV (Sigma), respectively, in PBS containing 1% bovine serum albumin overnight at 4°C. Podocytes cultured on coverslips were fixed with cold methanol: acetone (1∶1) for 10 minutes at −20°C and blocked with 2% bovine serum albumin in PBS for 30 minutes. Cells were then incubated with the specific primary antibodies against podocin and WT1. Sections were washed in PBS extensively before incubated for 1 hour with affinity-purified secondary antibodies (Santa Cruz) at a dilution of 1∶100 in PBS containing 1% BSA. As a negative control, the primary antibody was substituted with nonimmune IgG. Random samples were selected and double stained with DAPI (4′,6′-diamidino-2- phenylindole, HCL) to visualize the nuclei. Slides were viewed and photographed with an Eclipse 80i epifluorescence microscope equipped with a digital camera (Nikon). Intensity of fluorescence was analyzed using the NIH Image program.

### Western blot Analysis

The kidney was homogenized and podocytes were lysed with RIPA buffer that contained 1% NP40, 0.1% SDS, 100μg/ml PMSF, 1% protease inhibitor cocktail and 1% phosphatase I and II inhibitor cocktail (Sigma) in PBS on ice. The supernatants were collected after centrifugation at 13,000× *g* at 4°C for 20 minutes. After protein concentration was determined using a Bio-Rad protein assay kit (Bio-Rad Laboratories, Hercules, CA), the lysate was mixed with an equal amount of 2 × SDS buffer. Samples were heated at 100°C for 5–10 minutes and frozen at −20°C for preparation. Sample aliquots (80μg/lane) were separated 8% or 10% SDS-polyacrylamide gels (Bio-Rad, Hercules, CA) and electrotransferred to a nitrocellulose membrane (Amersham Life Science Inc., Arington Heights, Illinois, USA). Blocked for1 hour a room temperature with 5% nonfat milk in TBS buffer, the membrane were then incubated for 16 hours at 4°C with various primary antibodies in TBS buffer supplemented with 5% milk. The podocin (P0372), WT1 (SAB2104329) and β-actin (A2066) antibodies were acquired from Sigma-Aldrich. The UCP2 (C-20) (sc-6525) was acquired from Santa Cruz. After binding of primary antibodies, the membranes were incubated with the secondary horseradish peroxidase conjugated IgG in 5% nonfat milk. The signals were visualized by enhanced chemiluminescence (ECL, Amersham Life Sciences Inc.).

### Reverse Transcriptase-Polymerase Chain Reaction (RT-PCR) Analysis

Total RNA was extracted using TRIzol RNA isolation system according to the instructions by the manufactured (Invitrogen). Briefly, the first strand cDNA synthesized by using a Reverse Transcription System (Promega, Madison, WI) with random primers at 42°C for 30 minutes. Polymerase chain reaction (PCR) was carried out by using a standard PCR kit on 1μl aliquots of cDNA and HotStarTaq Polymerase (Qiagen, Valencia, CA) with specific primer pairs for mouse UCP2 and GAPDH. The sequences of the primers were as follows: UCP2, 5′-CTCA GAAAGGTGCCTCCCGA-3′ (sense) and 3′-ATCGCCTCCCCTGTTGATGTGGTCA-5′ (anti- sense); GAPDH, 5′ GGTGAAGGTCGGTGTGAACG-3′ and 3′-CTCGCTCCTGGAAGATGG TG- 5′ (anti-sense). The PCR protocol consisted of 35 cycles at 94°C for 30 seconds, 57°C for 30 seconds and 72°C for 1 minute, followed by a final extension step at 72°C for 10 minutes. PCR products were size fractionated on agarose gels and detected by NA-green (D0133, Beyotime) staining.

### Albumin Influx Assay

A simple albumin influx assay was adapted to evaluate the filtration barrier function of podocyte monolayer, as described previously [39], [40], [41]. To evaluate the filtration barrier function of podocyte monolayer, podocytes (5×103) under differentiating conditions were seeded on the collagen-coated transwell filters (3μm pore; Corning) at the top chamber for 10 days. Podocytes were then changed to serum-free and glucose-free medium overnight. Pretreated with or without genipin for 30min, podocytes then treated with or without 30mM glucose for 48 hours. Cells were washed twice with PBS supplemented with 1mM MgCl2 and 1mM CaCl2 to preserve the cadherin-based junctions. The top chamber was then refilled with serum-free and glucose-free medium and the bottom chamber was filled with medium supplemented with 40mg/ml of bovine serum albumin. The podocytes were incubated at 37°C for 6 hours and the aliquot of medium from top chamber was collected for albumin concentration measurement by using a Bio-Rad protein assay kit (Bio-Rad Laboratories, Hercules, CA).

### Statistical analysis

Data collected were expressed as mean ± SEM. Western blot and immunofluorescent staining were performed at least three times independently. Western blot analysis was completed by scanning and analyzing the intensity of hybridization signals using the NIH Image program. Statistical analyses of data were performed using Sigma Stat software (Jandel Scientific Software). Comparison between groups was made using one-way ANOVA, followed by the *t* test. *P<*0.05 was considered significant.

## References

[1] Liu S, Wang W, Zhang J, He Y, Yao C (2002). Prevalence of diabetes and impaired fasting glucose in Chinese adults, China National Nutrition and Health Survey.. Prev Chronic Dis.

[2] Rajpathak SN, Wylie-Rosett J (2011). High prevalence of diabetes and impaired fasting glucose among Chinese immigrants in New York City.. J Immigr Minor Health.

[3] Lee JW, Brancati FL, Yeh HC (2011). Trends in the prevalence of type 2 diabetes in Asians versus whites: results from the United States National Health Interview Survey, 1997–2008.. Diabetes Care.

[4] Lu B, Yang Z, Wang M, Yang Z, Gong W (2011). High prevalence of diabetic neuropathy in population-based patients diagnosed with type 2 diabetes in the Shanghai downtown.. Diabetes Res Clin Pract.

[5] Yang W, Lu J, Weng J, Jia W, Ji L (2011). Prevalence of diabetes among men and women in China.. N Engl J Med.

[6] Zhang PP, Ge YC, Li SJ, Xie HL, Li LS (2011). Renal biopsy in type 2 diabetes: timing of complications and evaluating of safety in Chinese patients.. Nephrology (Carlton).

[7] Langham RG, Kelly DJ, Cox AJ, Thomson NM, Holthofer H (2002). Proteinuria and the expression of the podocyte slit diaphragm protein, nephrin, in diabetic nephropathy: effects of angiotensin converting enzyme inhibition.. Diabetologia.

[8] White KE, Bilous RW, Marshall SM, El Nahas M, Remuzzi G (2002). Podocyte number in normotensive type 1 diabetic patients with albuminuria.. Diabetes.

[9] Wolf G, Chen S, Ziyadeh FN (2005). From the periphery of the glomerular capillary wall toward the center of disease: podocyte injury comes of age in diabetic nephropathy.. Diabetes.

[10] Dai C, Yang J, Bastacky S, Xia J, Li Y (2004). Intravenous administration of hepatocyte growth factor gene ameliorates diabetic nephropathy in mice.. J Am Soc Nephrol.

[11] Campbell KN, Raij L, Mundel P (2011). Role of angiotensin II in the development of nephropathy and podocytopathy of diabetes.. Curr Diabetes Rev.

[12] Mathieson PW (2009). Update on the podocyte.. Curr Opin Nephrol Hypertens.

[13] Toyoda M, Najafian B, Kim Y, Caramori ML, Mauer M (2007). Podocyte detachment and reduced glomerular capillary endothelial fenestration in human type 1 diabetic nephropathy.. Diabetes.

[14] Susztak K, Raff AC, Schiffer M, Bottinger EP (2006). Glucose-induced reactive oxygen species cause apoptosis of podocytes and podocyte depletion at the onset of diabetic nephropathy.. Diabetes.

[15] Dai C, Stolz DB, Kiss LP, Monga SP, Holzman LB (2009). Wnt/beta-catenin signaling promotes podocyte dysfunction and albuminuria.. J Am Soc Nephrol.

[16] Wharram BL, Goyal M, Wiggins JE, Sanden SK, Hussain S (2005). Podocyte depletion causes glomerulosclerosis: diphtheria toxin-induced podocyte depletion in rats expressing human diphtheria toxin receptor transgene.. J Am Soc Nephrol.

[17] Deedwania P (2011). Hypertension, dyslipidemia, and insulin resistance in patients with diabetes mellitus or the cardiometabolic syndrome: benefits of vasodilating beta-blockers.. J Clin Hypertens (Greenwich).

[18] Executive summary: standards of medical care in diabetes –2011.. Diabetes Care.

[19] Baker MK, Simpson K, Lloyd B, Bauman AE, Singh MA (2011). Behavioral strategies in diabetes prevention programs: a systematic review of randomized controlled trials.. Diabetes Res Clin Pract.

[20] Buse JB (2011). Type 2 diabetes mellitus in 2010: individualizing treatment targets in diabetes care.. Nat Rev Endocrinol.

[21] Deb DK, Sun T, Wong KE, Zhang Z, Ning G (2011). Combined vitamin D analog and AT1 receptor antagonist synergistically block the development of kidney disease in a model of type 2 diabetes.. Kidney Int.

[22] Gao Q, Shen W, Qin W, Zheng C, Zhang M (2011). Treatment of db/db diabetic mice with triptolide: a novel therapy for diabetic nephropathy.. Nephrol Dial Transplant.

[23] Okada T, Wada J, Hida K, Eguchi J, Hashimoto I (2006). Thiazolidinediones ameliorate diabetic nephropathy via cell cycle-dependent mechanisms.. Diabetes.

[24] Zhang CY, Parton LE, Ye CP, Krauss S, Shen R (2006). Genipin inhibits UCP2-mediated proton leak and acutely reverses obesity- and high glucose-induced beta cell dysfunction in isolated pancreatic islets.. Cell Metab.

[25] Zhang CY, Baffy G, Perret P, Krauss S, Peroni O (2001). Uncoupling protein-2 negatively regulates insulin secretion and is a major link between obesity, beta cell dysfunction, and type 2 diabetes.. Cell.

[26] Qian Y, Feldman E, Pennathur S, Kretzler M, Brosius FC 3rd (2008). From fibrosis to sclerosis: mechanisms of glomerulosclerosis in diabetic nephropathy.. Diabetes.

[27] Schena FP, Gesualdo L (2005). Pathogenetic mechanisms of diabetic nephropathy.. J Am Soc Nephrol.

[28] Wolf G, Ritz E (2003). Diabetic nephropathy in type 2 diabetes prevention and patient management.. J Am Soc Nephrol.

[29] Ziyadeh FN, Sharma K (2003). Overview: combating diabetic nephropathy.. J Am Soc Nephrol.

[30] Haneda M, Koya D, Isono M, Kikkawa R (2003). Overview of glucose signaling in mesangial cells in diabetic nephropathy.. J Am Soc Nephrol.

[31] Mason RM, Wahab NA (2003). Extracellular matrix metabolism in diabetic nephropathy.. J Am Soc Nephrol.

[32] Dalla Vestra M, Masiero A, Roiter AM, Saller A, Crepaldi G (2003). Is podocyte injury relevant in diabetic nephropathy? Studies in patients with type 2 diabetes.. Diabetes.

[33] Zheng S, Carlson EC, Yang L, Kralik PM, Huang Y (2008). Podocyte-specific overexpression of the antioxidant metallothionein reduces diabetic nephropathy.. J Am Soc Nephrol.

[34] Davis B, Dei Cas A, Long DA, White KE, Hayward A (2007). Podocyte-specific expression of angiopoietin-2 causes proteinuria and apoptosis of glomerular endothelia.. J Am Soc Nephrol.

[35] Eremina V, Cui S, Gerber H, Ferrara N, Haigh J (2006). Vascular endothelial growth factor a signaling in the podocyte-endothelial compartment is required for mesangial cell migration and survival.. J Am Soc Nephrol.

[36] Godel M, Hartleben B, Herbach N, Liu S, Zschiedrich S (2011). Role of mTOR in podocyte function and diabetic nephropathy in humans and mice.. J Clin Invest.

[37] Dai C, Stolz DB, Bastacky SI, St-Arnaud R, Wu C (2006). Essential role of integrin-linked kinase in podocyte biology: Bridging the integrin and slit diaphragm signaling.. J Am Soc Nephrol.

[38] Yang J, Liu Y (2001). Dissection of key events in tubular epithelial to myofibroblast transition and its implications in renal interstitial fibrosis.. Am J Pathol.

[39] Bu X, Zhou Y, Zhang H, Qiu W, Chen L (2011). Systemic administration of naked plasmid encoding HGF attenuates puromycin aminonucleoside-induced damage of murine glomerular podocytes.. Am J Physiol Renal Physiol.

[40] Li Y, Kang YS, Dai C, Kiss LP, Wen X (2008). Epithelial-to-mesenchymal transition is a potential pathway leading to podocyte dysfunction and proteinuria.. Am J Pathol.

[41] Rico M, Mukherjee A, Konieczkowski M, Bruggeman LA, Miller RT (2005). WT1-interacting protein and ZO-1 translocate into podocyte nuclei after puromycin aminonucleoside treatment.. Am J Physiol Renal Physiol.

